# Development of the Clinical Insight Questionnaire: A Novel Clinical Tool for the Assessment of Insight Into Cognitive Symptoms and Everyday Functioning in People With Neurodegeneration

**DOI:** 10.1002/gps.70165

**Published:** 2025-10-28

**Authors:** Catherine Pennington, Harriet Ball, Peter Connelly, Elizabeth Coulthard, Gordon Duncan, Chineze Ivenso, Tobias Langheinrich, Vivek Pattan, Terence Quinn, Karen Ritchie, Craig Ritchie, Tom Russ

**Affiliations:** ^1^ University of Edinburgh & NHS Lothian Edinburgh UK; ^2^ NHS Forth Valley Larbert UK; ^3^ University of Bristol Bristol UK; ^4^ Neuroprogressive Disorders and Dementia Research Network Dundee UK; ^5^ Aneurin Bevan University Health Board Glasgow UK; ^6^ Greater Manchester Neuroscience Centre Salford UK; ^7^ University of Manchester Manchester UK; ^8^ University of Glasgow Glasgow UK; ^9^ French Institute of Health and Medical Research Montpellier France; ^10^ Scottish Brain Sciences Edinburgh UK

**Keywords:** anosognosia, dementia, insight, mild cognitive impairment, neurodegeneration, symptom awareness

## Abstract

**Objectives:**

Altered insight into cognitive symptoms and diagnosis is a common feature of neurodegeneration, and can adversely impact on quality of life and ability to access medical care. Affected individuals can lose awareness of their symptoms and therefore decline to engage with medical assessment and treatment. Assessing insight is difficult, and there is a lack of short, easily administered clinical assessment tools. The aim of this study was to develop and evaluate the feasibility of a novel insight assessment questionnaire (The Clinical Insight Questionnaire, CLIQ) which can be independently completed by adults with a range of cognitive abilities.

**Methods:**

A discrepancy score approach was used to evaluate insight. A novel questionnaire targeting the domains of memory, personality and social behaviour, executive function, language, and activities of daily living was devised using the Delphi approach and public feedback. Participant and informant mirror versions of each item were written. The discrepancy between participant and informant scores provides an overall insight score. 12 UK based experts in cognitive disorder diagnosis and assessment were invited to review potential questionnaire items, as was a PPI group. A feasibility study was conducted where people with mild memory or thinking symptoms and an informant completed the questionnaire and the Montreal Cognitive Assessment.

**Results:**

Following an iterative process using the expert Delphi group and public feedback, 20 final questionnaire items were selected from an initial pool of 30 items. 21 people with mild cognitive symptoms but no formally diagnosed cognitive disorder (median MoCA score 24.5) participated in feasibility testing. The mean discrepancy score was 1.14, close to the ideal score of zero. No participants found the assessment upsetting or too long, and 81% rated the questions as easy to understand.

**Conclusions:**

The Clinical Insight Questionnaire (CLIQ) is a novel clinical tool for the assessment of insight in people with mild to moderate neurodegeneration. In feasibility testing it was quick and easy for people with mild cognitive symptoms and informants to self‐complete. Initial feasibility testing showed very promising findings for usability and acceptability, and a full validation study is now in progress.

## Key Points

- Insight into symptoms should be formally evaluated in cognitive clinics.
- Using a structured assessment tool may help with the clinical assessment of insight.
- The Clinical Insight Questionnaire (CLIQ) is designed for clinical use and is accessible for use by people with mild cognitive problems.

## 1 Introduction

There are an estimated 885,000 people with dementia in the UK, a number predicted to nearly double by 2040 [1]. Altered insight into symptoms and abilities is frequently experienced by people with dementia [2, 3], and can also affect people with mild cognitive impairment (MCI) [4, 5, 6]. Definitions of insight vary [7], as reflected by the heterogeneous terminology in use, including ‘insight’, ‘symptom awareness’ and ‘anosognosia’ [7, 8]. Here we define insight as internal knowledge of one's abilities and symptoms. Insight is theorised to be object specific, for example, insight into memory is separate to insight into diagnosis [9, 10]. Measuring insight is inherently challenging, being an internal state. Findings are influenced by the definition of insight adopted, how it is measured and the object of interest [9]. Disruption of insight will, by definition, cause inaccurate reporting of one's insight ability [8]. Personality and cultural values may also influence insight in health and disease [11]. How far changes to insight are directly determined by neurodegeneration, and how much they are mediated by individual psychological factors is a matter of on‐going debate, and may vary between different forms of neurodegeneration and different individuals [3, 12, 13]. Cognitively healthy older adults tend to have a negative view of their cognition, despite being able to accurately predict their performance on individual cognitive tests [14]. Over‐estimation of cognitive ability in MCI may predict an increased risk of future dementia [5, 15, 16].

The type of neurodegenerative process influences the degree of insight loss and which objects are affected. Altered insight into symptoms is very common in Alzheimer's Disease dementia. Castrillo et al. found over 70% of individuals to have insight changes at the point of diagnosis, whilst Kalbe et al. evaluated a group of people with mild dementia, most of whom had altered insight [17, 18]. People with behavioural variant Frontotemporal Dementia typically show early, severe reduction of insight towards behavioural changes [2, 19].

Reduced insight into cognitive change may lead to delayed diagnosis, potentially cause issues with personal safety (e.g., continued driving or use of kitchen appliances when the individual cannot do so safely), and create barriers to accessing treatment and social support. Whilst various insight evaluation tools exist, the majority have been developed for research use and are not suitable for routine clinical use due to their length, terminology or the need for administration by a trained staff member. In routine clinical practice the insight assessment largely relies on clinician judgement following an unstructured interview with the individual and an informant [2, 13, 20]. Relatives, friends and carers of people with cognitive disorders often identify reduced insight. Frequently the clinical consultation is initiated by family or friends, and the affected individual may only report that other people are concerned about their memory and thinking (rather than being independently aware of cognitive symptoms), or only acknowledge trivial changes [21]. Altered insight may lead to individuals declining to attend cognitive assessments, despite significant cognitive decline reported by family and primary care [22].

Changes to insight may lead to safety issues in the home and place individuals at higher risk of financial exploitation [23], and consequently greater informal or formal care needs [24]. Reduced insight into symptoms can adversely affect quality of life for affected individuals and informal carers [2, 25], and increase the likelihood of an individual entering residential care [26, 27]. Conversely, individuals with dementia and reduced insight may report lower levels of anxiety and depression compared to people with a similar level of cognitive impairment but intact insight [28, 29]. Insight is therefore an important concept to evaluate clinically.

Factors that may influence insight include premorbid personality type and attitudes towards illness. Seiffer et al. [30] found that insight was influenced by dementia type, severity, duration and degree of anxiety and depression, but not by personality trait or avoidant coping. Previous studies have found people with dementia to be accurate in describing their premorbid personality, but to have reduced awareness of personality change in recent years [8]. The ‘petrified self’ model hypothesises that neurodegeneration impairs an individual's ability to remember how their abilities have changed, and hence they are unable to update their internal model of their cognitive and physical functioning [8]. The close family and friends of an individual with neurodegeneration may show denial or avoidance of symptoms [31]. The spouses of affected individuals report experiencing grief and needing time to accept the diagnosis and potential implications for changes in familial status—with the affected individual relinquishing roles they are unable to carry out, and family members becoming carers [32, 33].

Many insight evaluation tools utilise an informant report, which is compared to the participant's report with any discrepancy used to quantify changes to insight [20, 34]. Informant report is not an unbiased standard and may be influenced by factors such as carer stress [35]. An alternative is to compare self‐rated performance before, during or after a cognitive task with objective task scores, however this assumes that cognitively healthy adults have an optimal ability to self‐assess task performance, which is unlikely to be universally correct [36, 37]. Self‐assessment in healthy and cognitively impacted adults may theoretically be influenced by knowledge of the task, age and personality and mood factors [12, 38, 39]. Specific neuropsychological tasks have been developed to capture awareness of cognitive performance, such as Judgement of Learning and Feeling of Knowing tasks [40, 41, 42]. Such tasks tend to focus on recent episodic memory, and are not easily translated to a clinical environment.

Given how common altered insight is in neurodegeneration, there is a need for a clinical tool for insight evaluation that can be used in routine clinical practice. A range of scales have been published, but tend to either be too long and complex for routine clinical use, or have a narrow focus on limited object(s) of insight [13, 34]. We set out to create a short, accessible assessment tool suitable for use in clinical settings, with coverage of major cognitive domains and functional activities. The aim was to create a tool that could be self‐completed by a person with subjective, mild or moderate cognitive impairment and an informant. A Delphi group approach was selected as the most appropriate way to achieve expert consensus on suitable questionnaire items, and the resulting tool was then tested for feasibility with volunteers with mild memory and thinking symptoms. Priorities in design were to ensure that the tool was accessible to adults with cognitive impairment due to a variety of causes or modest baseline educational attainment, and that it could be quickly completed with minimal assistance. Subject to appropriate validation, we envisage that the CLIQ could be useful to demonstrate early insight changes in people with subjective or mild cognitive impairment as a potential diagnostic indicator of neurodegeneration, and to evaluate the severity of insight changes in people with clinical dementia.

## 2 Materials and Methods

### 2.1 Clinical Insight Questionnaire (CLIQ) Development

A modified Delphi approach was used [43], with the pre‐specified purpose of creating a novel discrepancy questionnaire for the assessment of insight in a clinical setting. We wished to cover a range of objects of insight, and create a questionnaire that could be self‐completed by someone with mild to moderate cognitive impairment with minimal or no external support. 30 initial items were generated, with a pre‐specified plan to select the 20 most suitable items for the final questionnaire. In the first round, questionnaire items underwent expert review. The feedback from the first Delphi round was used to refine and select items for the second draft of the CLIQ, which was then subject to a second Delphi round and public evaluation.

The initial panel of items were devised by three specialists in cognitive disorders (CP, KR, PC). The CLIQ items were devised to cover domains of memory, personality and social behaviour, executive function, language, and activities of daily living. A bank of questions was devised using a modified Likert approach, with respondents asked how often they identified with a statement such as ‘I ask the same question several times in a few hours’ with options of ‘never’, ‘rarely’, ‘sometimes’, ‘often’ or ‘always’. Mirror questions were created for informants. Questions were written for a reading stage of age 11–12 years (US 6^th^ grade, UK year 7).

Public and professional responses were collected using the Jisc Online Surveys tool. Public responses were collected online and remained anonymous throughout. The study was advertised to research volunteers registered with the Scottish Brain Health Register and Parkinson's UK. Participants were provided with background information about the CLIQ and data usage prior to completing the online form. As per NIHR PPI guidance [44], informed consent was not taken from members of the public providing feedback on draft forms of the CLIQ.

UK based clinical specialists in the assessment and diagnosis of cognitive disorders were approached to join the Delphi group. Thirty questions were presented in the initial draft of the CLIQ, spread equally across the five domains of interest. During the first round of the Delphi Group experts in cognitive disorders (4 consultants in old age psychiatry, 1 neuropsychologist, 5 neurology consultants or registrars and 2 care of the elderly consultants) were asked to select the four most useful questions in each category, and rate ease of reading. They were given free text options to suggest alternative questions and provide overall feedback. The CLIQ was then edited in line with their feedback and the most relevant 25 items retained.

Following the first round of expert review of the proposed items, the second draft of the CLIQ were reviewed by members of the public. Respondents were asked to rate the importance of assessing insight in a memory clinic setting. For each item they were asked *‘Would you feel upset, annoyed or angry if you were asked to respond to these questions?’* (Acceptability rating) and *‘Please select any questions you feel are NOT easy to read and understand.’* (Readability rating). A second round of expert review was undertaken of the second draft, and the CLIQ was then revised in line with results of profession and public feedback.The lowest scoring items from each domain section was removed. Final revisions of the CLIQ were made prior to the feasibility study.

### 2.2 Feasibility Testing of CLIQ

People aged 60 years or over with persistent, mild cognitive symptoms were invited to participate in a feasibility study of the CLIQ. People with or without a formal diagnosis of Mild Cognitive Impairment or Subjective Cognitive Decline were eligible. Exclusion criteria included: lack of English language fluency; hearing or visual impairment likely to confound study procedures; inability to give informed consent to study participation; current moderate or severe mental health disorders (including alcohol or substance abuse); use of sedative or cognitively toxic medication; people with marked daily variability in cognition or rapidly progressive cognitive decline; learning difficulties and/or very low/absent literacy; recent (within 4 months of recruitment) delirium, traumatic brain injury, stroke, neurosurgery or other major disturbance of brain functioning; unstable medical problems likely to affect cognition, or compromise study participation. Participants were recruited from Join Dementia Research, the Scottish Brain Health Register, and The Scottish Health Research Register & Biobank (SHARE). All participants and study partners provided informed consent to study participation. Each dyad completed a participant and informant version of the CLIQ, and provided feedback on it. The participant also completed the Montreal Cognitive Assessment (MoCA) [45].

The order of study procedures was as follows: informed consent; collection of demographic details; CLIQ completion; completion of CLIQ feedback form; MoCA administration. All study visits were in person. A paper copy of the CLIQ was read and completed independently. For participants with visual impairment the CLIQ was read aloud and responses documented by the researcher. The participant and informant were asked not to discuss their responses. They were encouraged to ask the researcher if any questions were unclear.

### 2.3 Scoring of the CLIQ

The lowest possible individual score is 20 and indicates the best possible functional rating. The highest possible score from either the participant or their informant is 100 and indicates the greatest functional impairment. The ‘ideal’ discrepancy score is zero. The lowest possible total discrepancy score is −80 (indicated marked ***over***‐estimation of functioning by the participant) and the highest possible discrepancy score is +80 (indicating marked ***under***‐estimation of functioning by the participant). It has been proposed [46, 47] that discrepancy scores should be adjusted to account for the overall level of impairment, as shown by the total score given by the participant and informant.

$$\text{Adjusted}\;\text{discrepancy}\;\text{score}=\frac{\left(\text{Participant}\;\text{score}—\text{Informant}\;\text{score}\right)}{\left((\text{Participant}\;\text{score}+\text{Informant}\;\text{score})/2\right)}$$

Applying this method to the current scale the ideal score is zero, the lowest score is −1.3 and the highest score is 1.3.

The study sponsor is ACCORD (www.accord.ed.ac.uk). Ethical approval was provided by the West of Scotland Research Ethics Committee 1 (REC reference 22/WS/0024). The methodology was approved by the aforementioned ethics committee and sponsor and REC requirements adhered to throughout.

## 3 Results

### 3.1 Clinical Insight Questionnaire (CLIQ) Development

Thirty‐four members of the public reviewed the second draft of the CLIQ. Their ratings of CLIQ items are shown in Table 1. Basic demographic information was collected for public respondents. 50% self‐described as having a medical condition affecting their memory or thinking, and a further 12.5% had memory or thinking symptoms but no medical diagnosis. 5.9% were between 41 and 50 years of age, 20.6% between 51 and 60, 29.4% between 61 and 70, 35.2% between 71% and 80% and 8.8% were over 80 years of age. When asked to rate the importance of assessing insight in a memory clinic setting, 72.7% rated this as ‘very important’. Readability and Acceptability ratings for each item are shown in Table 1. Healthcare professional ratings of items in the second draft of the CLIQ are shown in Table 1 (with a higher percentage indicating a higher number of professionals selected the item for inclusion in the final draft).

**TABLE 1:** Healthcare professional and Public review of Draft two of the Clinical Insight Questionnaire.

	Sub‐section	Professional review (% selecting each item)	Public review	
			Readability (%)	Acceptability (%)
I ask the same question several times in a few hours	Memory	100.0	57.1	40.0
I forget what happened in a TV programme soon after I watch it		66.7	57.1	70.0
I forget appointments		100.0	100.0	90.0
I find it easier to remember events from many years ago than events from a few weeks ago		100.0	57.1	90.0
My memory is worse than other people of my age*		33.3	71.4	30.0
I get irritated more easily than I used to	Personality and behaviour	100.0	40.0	44.4
I am less interested in social activities or conversations than I used to be		100.0	60.0	66.7
I get upset and cry more easily than I used to		66.7	100.0	44.4
I am less interested in things I used to enjoy		88.9	80.0	66.7
I am more anxious than I used to be*		44.4	40.0	66.7
I find it difficult to make plans	Executive Functioning	88.9	88.9	71.4
I find it difficult to make important decisions		77.8	88.9	42.9
I have problems managing my personal finances		100.0	100.0	42.9
I find it difficult to pay attention to 2 things at the same time such as having a conversation whilst watching a TV or driving*		66.7	44.4	57.1
I am less organised than I used to be		66.7	44.4	28.6
I struggle to find the right word to say	Language function	88.9	28.6	40.0
I struggle to understand what people say to me		88.9	100.0	60.0
I find it difficult to make myself understood in a conversation		88.9	57.1	60.0
My spelling and grammar are not as good as they used to be*		55.6	57.1	60.0
I struggle to follow a conversation with more than 1 person		77.8	71.4	40.0
I find it more difficult to manage my medication	Activities of daily living	88.9	80.0	33.3
I find it more difficult to use machines like the cooker, washing machine, microwave or dishwasher		100.0	60.0	50.0
I find it more difficult to use a mobile phone or computer		88.9	20.0	33.3
Other people are worried about my driving		66.7	60.0	16.7
I need help with washing or dressing*		55.6	80.0	33.3

* Asterixis indicate items which were removed following the second professional review.

**TABLE 2:** Feasibility study participant and informant characteristics.

Participant Characteristics		
Participant sex	13 female	8 male
Participant age	74 years (median) (IQR 70–82, range 60–84)	
Years of education	13 (median, IQR 11–10)	
Highest qualification	None	3 (14.3%)
	High school	4 (19.1%)
	Vocational	6 (28.6%)
	University	8 (38.1%)
MoCA	24.5 (median, IQR 23–26, range 14–29)^a^	
Informant characteristics		
Relationship to participant	Spouse	13
	Adult child	6
	Sibling	1
	Friend	1
Informant sex	15 female	6 male
Informant age	69.0 years (median) (IQR 53.5 to 76.8, range 42.0–83.0 years)	
Contact with participant	Daily	17 (81%)
	Weekly	2 (9.5%)
	Monthly	2 (9.5%)
Concerns over participant's cognition^b^	No concerns	6
	Ongoing concerns	14

^a^ One participant was excluded from the MoCA score results as they scored full marks and reported completing it very recently and recalling elements of it. For 3 participants a modified version of the MoCA was used due to visual impairment –the percentage scored was converted to a mark out of 30 for analysis purposes.

^b^ One informant did not complete this question. Of informants who reported concerns, 4 were significant concerns (e.g. ‘seems to be unable to remember most the conversations we have’) and 10 minor (e.g. ‘forgets names of people’).

### 3.2 Feasibility Study Results

Twenty‐one participant/informant dyads were recruited to the feasibility study. Participant and informant characteristics are shown in Table 2. Median MoCA score for participants was 24.5, whilst median years of education was 13. Five participants were prescribed at least 1 anticholinergic medication, and 6 were using potentially sedative analgesic medications. All participants had concerns about their cognition. Four had been assessed in a memory clinic but not received a specific cognitive diagnosis. Three people had a diagnosis of post‐stroke cognitive impairment, and one person had a diagnosis of transient epileptic amnesia. One person was awaiting a memory clinic appointment having been referred by their GP with suspected dementia (this was not disclosed by the participant prior to study assessment). There were no participants with a dementia diagnosis at the time of study procedures. Five participants reported alcohol use above current government recommended safe limits (3 between 14 and 30 units a week, one person 49 units, one person 70 units). No additional participants reported previous heavy alcohol use.

Overall, most participants gave positive feedback about the CLIQ. The mean time for participants to complete the CLIQ was 3 min and 44 s (range 2–9 min). Only 4 (19%) found the questions difficult to understand, and no participants found the questions upsetting. 2 (10%) participants had visual impairment and the CLIQ was completed verbally. All other participants and informants completed the CLIQ independently. 7 (33%) of participants did not complete all questionnaire items, however in 5 instances this was because a question regarding driving ability was not relevant as they did not drive (or had stopped driving for non‐cognitive medical reasons). Free text comments were encouraged—the majority noted when an item was not relevant to the participant (e.g., a driving ability question to a non‐driver), or provide information on strategies used to support an activity. 3 (14%) informants and 1 (5%) participant felt choosing between the ratings was difficult. No participants rated the CLIQ as being too long, whilst 2 (10%) informants rated it as too short.

**FIGURE 1:**
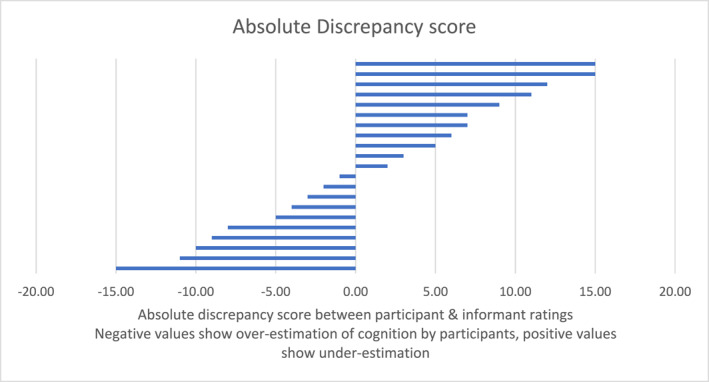
Absolute discrepancy scores (group mean 1.14, standard deviation 8.91).

**FIGURE 2:**
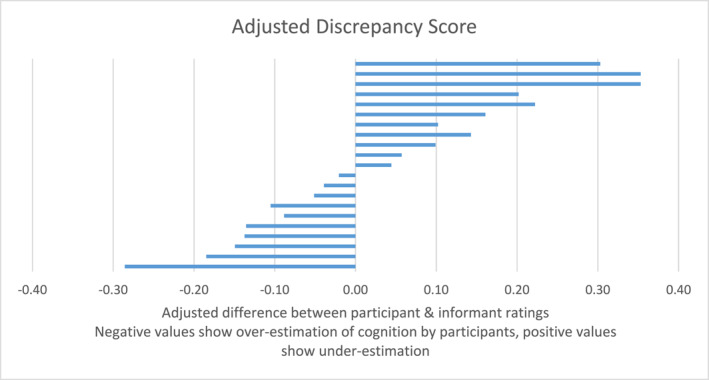
Adjusted discrepancy scores (mean 0.04, standard deviation 0.18).

Raw and adjusted scores are shown in Figures 1 and 2. From the overall scores (where the ideal discrepancy score is zero, with a possible range from −80.0 to 80.0), 10 participants had a positive discrepancy score (mean 8.36) and 11 had a negative score (mean −6.80). The overall group mean discrepancy score was 1.14 (standard deviation 8.91). Using the adjusted score method (where the ideal score is zero, the lower −1.3 and the highest possible is 1.3), the mean positive adjusted discrepancy score was 0.19 and the mean negative score was −0.12. The overall mean adjusted discrepancy score was 0.04 (standard deviation 0.18). Looking at either the raw or adjusted score, the results were close to the ideal score. As expected, the discrepancy scores in this feasibility group of older adults with mild self‐reported cognitive symptoms was close to zero, indicating a good level of symptom awareness.

## 4 Discussion

Here we describe the development of a practical, quick and easy to use tool for assessing insight in people with neurodegeneration in a clinical setting. The CLIQ was designed to require minimal instruction from staff, and during feasibility testing typically took less than 5 min to complete. Feedback from participant/informant dyads was positive, with some caveats around the inherently subjective nature of the Likert rating scale. No participants or informants showed any distress or upset due to CLIQ items. The CLIQ was feasible for self‐completion by adults with mildly impaired cognition and average educational attainment. CLIQ scores showed no or mild changes to insight, as would be predicted for this group.

Studies of altered insight in people with MCI show linkage with altered metabolism in the posterior cingulate cortex and hippocampus [48], and people with amnestic MCI and impaired insight are at higher risk of progression to dementia compared to those with intact insight [49]. This suggests that altered insight is an early biological consequence of the neurodegenerative process. There is an increasing emphasis on the early diagnosis of dementia [50, 51] to facilitate appropriate symptomatic management, and allow affected individuals to participate more fully in health and welfare decision making, and planning their own future [52]. This is also likely to increase the volume of people seeking medical assessment of mild cognitive symptoms. Mild cognitive changes have multiple potential non‐neurodegenerative causes, and assessing early insight changes could be a valuable part of the assessment of individuals with mild cognitive symptoms. Insight assessment is also essential for adults with dementia, due to the potential impacts on personal autonomy, safety and quality of life.

Limitations of the current work are lack of information regarding test‐retest reliability, the influence of carer stress, and ease of use for people with moderate to severe dementia. The next stage of this work is a validity study which will address these issues. Informant report is a potentially unreliable standard—it depends on informants having sufficient direct contact with the participant, being able to accurately observe their abilities, and being free from conscious or subconscious bias [53, 54]. This is not unique to insight assessment and can affect any part of the memory clinic evaluation, where informant reports are heavily used. It is difficult to work around this, and clinicians should always be mindful of the possibility of inaccurate informant reports. Other techniques to assess insight which do not involve informant report exist, but use complex neuropsychological methods such as Feeling of Knowing tasks which are impractical in clinical services. The Healthcare Awareness Profile Inventory has been recently developed to evaluate insight in dementia, and is awaiting validation [55]. There is good evidence that insight tends to worsen as dementia progresses, but it is unclear if this happens in a linear fashion [2, 5, 56]. Evaluating insight becomes more challenging in people with more advanced dementia, due to the severity of changes to memory and language [57]. The CLIQ has been designed for people with MCI, mild or moderate dementia, and whilst we plan to trial it in people with severe dementia, it is likely to be less appropriate for this group. Changes to insight have different diagnostic and therapeutic implications at different stages of cognitive decline and in different forms of neurodegeneration. If impaired insight is coupled with amnestic MCI, this is suggestive of increased risk of future dementia. In mild to moderate dementia, reduced insight should prompt detailed exploration of home safety, particularly around ability to self‐determine financial and driving safety. In certain clinical scenarios, early and severe impairment of insight can contribute to a specific diagnosis (e.g., behavioural variant frontotemporal dementia).

Failing to systematically evaluate insight in clinical settings can lead to misdiagnosis, particularly for individuals with early or prodromal dementia and well‐preserved social behaviours. It is therefore essential to assess insight in a systematic and robust fashion in all individuals presenting to cognitive services. Use of a structured assessment can help highlight areas which should be explored in greater detail during clinical history taking. Informants also have a neutral opportunity to provide information they may not feel able to disclose in front of their loved one. Whilst many tools to evaluate insight have been published, these skew towards usage in research settings and typically cannot be completed independently. The CLIQ is designed to be a quick, easy to use tool to aid in the assessment of insight in the cognitive clinic. We envisage that the CLIQ could be completed independently prior to a cognitive clinic appointment, providing the clinician with additional information to aid their differential diagnosis. The CLIQ is not intended to be used in isolation, but as part of a comprehensive cognitive assessment. It has been designed with consideration of typical insight changes seen in common forms of dementia, but subject to appropriate validation may also be useful in other scenarios such as traumatic brain injury. We are currently conducting a larger validity study recruiting healthy controls, people with a diagnosis of MCI and people with mild dementia to establish the validity of the CLIQ and investigate correlations with performance in different cognitive domains.

## Conflicts of Interest

The authors declare no conflicts of interest.

## Supporting information


Supporting Information S1

## Data Availability

Anonymised summary responses are available by contacting the lead author.
