# Supplementary material for: Development of the Clinical Insight Questionnaire: A Novel Clinical Tool for the Assessment of Insight Into Cognitive Symptoms and Everyday Functioning in People With Neurodegeneration
Source: Int J Geriatr Psychiatry. 2025 Oct 28;40(11):e70165. doi: 10.1002/gps.70165 (PMC12569398; doi:10.1002/gps.70165)
Supplement: Supplementary file 1 — Supporting Information S1 [file GPS-40-e70165-s001.docx]

**Title: Development of the Clinical Insight Questionnaire: a novel clinical tool for the assessment of insight into cognitive symptoms and everyday functioning in people with neurodegeneration**

**APPENDIX**

Flowchart of profession and public input to development of the CLIQ.

*The original 30 items were (written by CP, KR, PC)

Public Review

Delphi group

Round 2

Final 20 items selected

Questionnaire Revision: 25 items selected

Delphi group

Round 1

30 question items*

**Participant freetext comments on the Clinical Insight Questionnaire:**

| It didn't cover the development of memory loss, otherwise excellent |
| --- |
| Found it pretty straightforward |
| The difference between 'rarely' and 'sometimes' is quite difficult to gauge when completing the questionnaire. I probably marked 'sometimes' if I could think of something happening more than once recently. |
| I enjoyed the challenge |
| It was easy to understand but the answers were not clear |
| Not sure of answer to Q1 |
| Easy to understand |
| Needs help to fill in forms |
| I was anxious to make my answers helpful |
| You did not seem to mention loss of hearing or sight causing problems. |
| Difficult to put into context or translate to my individual situation. |

**Participant Clinical Insight Questionnaire feedback:**

| **Clinical Insight Questionnaire length** | |
| --- | --- |
| Too short | 0 |
| About right | 21 |
| Too long | 0 |
| **Questions difficult to understand** | |
| Yes | 4 |
| No | 17 |
| **Questions upsetting** | |
| Yes | 0 |
| No | 21 |

**Informant free text comments on the Clinical Insight Questionnaire:**

| I found some of the questions mildly upsetting because they point ahead to what might happen. I felt I wanted to add qualifiers to some of the answers or offer a specific example. No questions about remembering places or having visited places - my husband has noticeable memory gaps in having visited somewhere - big change to previously |
| --- |
| I found the questionnaire easy to follow |
| Sometimes confusing to have the 'never, rarely' et response of 1-10 score. Would be easier to answer 1-5 or 1-10 could be more accurate. |
| Would be helpful to have some space on each page to write additional comments eg Q12 I would add that it took quite a bit longer to be organised, Q13 & 14 would add that he wears hearing aids and difficulties were more due to not hearing properly in certain situations |
| I would think there could be room for more questions if needed. It was clear and easy to complete quickly so some more time could be spent. Some questions a 'does not apply' might be helpful (eg driving, my mother hasn't driven for 30 years) |
| Easy to answer. Feels quite vague though. No questions about ie losing track of a topic of conversation in the middle of speech; changes to personality or behaviour |
| form filling is an issue so put that in as a question |
| The difference between 'sometimes' & 'often' answers is difficult to ascertain for some questions. |
| Some of the answers to the questions reflected problems that I have (due to 'MS' fog) rather than to his abilities |

**Informant Clinical Insight Questionnaire rating:**

| **Clinical Insight Questionnaire length*** | |
| --- | --- |
| Too short | 2 |
| About right | 18 |
| Too long | 0 |

* One informant did not answer this question.
